# Phenotypic and genotypic detection of virulence factors of *Staphylococcus aureus* isolated from clinical and subclinical mastitis in cattle and water buffaloes from different farms of Sadat City in Egypt

**DOI:** 10.14202/vetworld.2015.1051-1058

**Published:** 2015-09-08

**Authors:** Mohamed Sabry Elsayed, Abd Elrahman Mahmoud El-Bagoury, Mai Abdallah Dawoud

**Affiliations:** 1 Department of Bacteriology, Mycology and Immunology Faculty of Veterinary Medicine University of Sadat City, Menoufia 32897, Egypt; 2 Department of Food Hygiene and Control, Faculty of Veterinary Medicine University of Sadat City, Menoufia, 32897, Egypt; 3 Library of Faculty of Veterinary Medicine, University of Sadat City, Menoufia, 32897, Egypt

**Keywords:** clinical and subclinical mastitis, enterotoxins, identification, *Staphylococcus aureus*, polymerase chain reaction, virulence factors

## Abstract

**Aim:**

To characterize *Staphylococcus aureus* from clinical and subclinical mastitis and identify virulence factors.

**Materials and Methods:**

Two hundred and two milk samples were collected, 143 from mastitic cattle and buffaloes 94 and 49, respectively, and 59 from apparently healthy cattle and buffaloes 35 and 24, respectively.

**Results:**

California mastitis test was applied and positive prevalence were 91.48% and 75.51% for cattle and buffalo with clinical mastitis and 37.14% and 45.83% for cattle and buffalo with subclinical mastitis. *S. aureus* was isolated from clinically mastitic cattle and buffaloes were 39.29% and 50%, respectively. While, from subclinical mastitic cattle and buffaloes were 80% and 72.73%, respectively. Hemolytic activity was assessed for *S. aureus* isolated from clinically and subclinical mastitic cases with prevalences of 100% and 56.25%, respectively. Thermo nuclease production from clinically and subclinical mastitic cases was 25% and 56.25%, respectively. Simplex polymerase chain reaction (PCR) conducted on *S. aureus* using 16S rRNA, clumping factor A, Panton valentine leukocidin, coagulase (Coa), alpha-hemolysin and beta-hemolysin those proved existence in 100%, 46.9%, 65.6%, 100%, 34.4%, and 43.75% of the isolates, respectively. While, multiplex PCR is utilized for detection of enterotoxins and proved that 12.5% was positive for enterotoxine Type D.

**Conclusions:**

It is concluded that simplex and multiplex PCR assays can be used as rapid and sensitive diagnostic tools to detect the presence of *S. aureus* and characterize its virulence factors that help in detection of severity of infection, distribution and stating preventive and control strategies.

## Introduction

Verily, *Staphylococcus aureus* is prevalent worldwide as a pathogen causing intra-mammary infections (IMI) in dairy cows and thus of economic significance to milk producing dairy farms, as it reduces milk quality and production. It has been found responsible for more than 80% of subclinical bovine mastitis, which may result in about $300 loss per animal [1]. Virulence factors of *S. aureus* are vital for the pathogenesis as well as for diagnosis of *S. aureus*. Clumping factor A (*CLFA*) is considered one of essential adhesion factors and has been identified as a virulence factor in an endocarditis model [2]. The role of coagulase (Coa) in IMI remains uncertain, but it is known that most *S. aureus* strains isolated from such infections in a matter of fact, coagulate bovine plasma [3]. Furthermore, *S. aureus* produces wide variety of exoproteins, including hemolysins that contribute to their ability to colonize and induce disease in mammalian hosts. Alpha hemolysin is cytotoxic while Beta-hemolysin is a sphingomyelinase expressed by the majority of strains isolated from IMI in bovines. It has been stated that both toxins increase *S. aureus* adhesion to epithelial cells of mammary gland [4,5].

*S.aureus* can produce one or more additional exoproteins, including toxic shock syndrome toxin-1 (TSST-1), staphylococcal enterotoxins A to U (*SEA*, *SEB*, *SEC*, *SED*, *SEE*, *SEG*, *SEH*, *SEI*, and *SEJ*), the exfoliative toxins (ETA and ETB), and Panton valentine leukocidin (PVL). These toxins play a prominent role in staphylococcal food poisoning and other types of infections in humans and animals. PVL is cytotoxin that leads to leukocyte destruction and tissue necrosis [6].

Due to the limitations of cultural methods, the development of polymerase chain reaction (PCR)-based methods the simplex and multiplex PCR assays can be used as a rapid and sensitive diagnostic tool to detect the presence of *S. aureus*, provide a promising option for the rapid identification made in hours, rather than days consumed by conventional cultural methods [7].

So, this study aimed to characterize *S. aureus* from clinically and subclinically mastitic cattle and buffaloes as well as detect some virulence factors such as hemolysins, thermo nuclease production, *CLFA*, PVL, Coa, alpha-hemolysin (Hla), Beta-hemolysin (Hlb), and enterotoxins.

## Materials and Methods

### Ethical approval

Ethical approval from cattle and buffalo’ owners and assurance of anonymity, witnessed by a veterinarian from the Egyptian Veterinary Medicine Authority was obtained.

### Animals and samples

202 milk samples were collected as follows 143 from mastitic cattle and buffaloes 94 and 49, respectively and 59 from apparently healthy cattle and buffaloes 35 and 24, respectively, 20 ml of milk samples were collected aseptically after cleaning of udder and throwing the first milk strips.

1. Milk samples were tested by California mastitis test (CMT) [8].
2. Isolation of *S. aureus* according using Mannitol salt agar, Baird-Parker agar media, Vogel Johnson agar and brain heart infusion broth.
3. Identification and biochemical characterization of *S. aureus* [9].
4. Confirmation of *S. aureus* and detection of some virulence factors using PCR.
  - DNA extraction: DNA of *S. aureus* was extracted for PCR analysis. For this purpose, *S. aureus* isolates were cultured in Mueller-Hinton broth overnight. Bacterial cells were collected by centrifugation for 10 min 5,000 rpm and washed in 1 mL of TE buffer (10 mM Tris HCl pH 8.0; 1 mM ethylenediaminetetraacetic acid) and recentrifuged for 10 min 14,000 rpm. Fifty microliters of lysostaphin (100 μg/mL) was added to the pellet, incubated for 10 min at 37°C and subsequently treated with 50 μL proteinase K (100 μg/ml) for 10 min at 37°C. For inactivation of proteinase K, the suspension was heated for 10 min at 100°C. Isolated DNA samples were kept at −20°C until further use [10].
  - Specific primers are targeting genes of 16 S rRNA, PVL, CLFA, Coa, Hla, Hlb, and enterotoxins, nucleotide sequences as well as product size (Table-1).

**Table-1:** List of primers, accession number and PCR product size.

Primers	Nucleotide sequence	Accession no.	PCR product size (bp)	Reference
(1) 16 S *rRNA*	16S rRNA F GTA GGT GGC AAG CGT TAT CC 16S rRNA R CGC ACA TCA GCG TCA G	KM507158.1	228	[11]
(2) *PVL*	PVL-F ATCATTAGGTAAAATGTCTGGACATGATCCA PVL-RGCATCAASTGTATTGGATAGCAAAAGC	HM584707.1	433	[12]
(3) *CLFA*	ClfA-F GGC TTC AGT GCT TGT AGG ClfA-R TTT TCA GGG TCA ATA TAA GC	KJ001294.1	980	[13]
(4) *Coa*	Coa –F CGA GAC CAA GAT TCA ACA AG Coa -R AAA GAA AAC CAC TCA CAT CA	KJ746934.1	Polymorphism 970, 910, 740, 410	[14]
(5) *Hla*	HLA-1CTGATTACTATCCAAGAAATTCGATTG HLA-2CTTTCCAGCCTACTTTTTTATCAGT	M90536	209	[15]
(6) *Hlb*	HLB-1 GTGCACTTACTGACAATAGTGC HLB-2 GTTGATGAGTAGCTACCTTCAGT	S72497	309	[15]
(7) Enterotoxins				
*SA-U* (20)	5’-TGTATGTATGGAGGTGTAAC-3’ Universal forward primer	-	-	[16]
*SA-A* (18)	5’-ATTAACCGAAGGTTCTGT-3’ Reverse primer for sea	GQ859135.1	270	
*SA-B* (18)	5’-ATAGTGACGAGTTAGGTA-3’ Reverse primer for seb	KC428707.1	165	
*SA-C* (20)	5’-AAGTACATTTTGTAAGTTCC-3’ Reverse primer for sec	GQ461752.1	69	
*ENT-C* (25)	5’-AATTGTGTTTCTTTTATTTTCATAA-3’ Reverse primer for sec	KF386012.1	102	
*SA-D* (20)	5’-TTCGGGAAAATCACCCTTAA-3’ Reverse primer for sed	CP007455.1	306	
*SA-E* (16)	5’-GCCAAAGCTGTCTGAG-3’ Reverse primer for see	EU604545.1	213	

Hlb=Betahemolysin, Hla=Alphahemolysin, Coa=Coagulase, CLFA=Clumping factor A, PVL=Panton valentine leukocidin, PCR=Polymerase chain reaction
  - Protocol and program of each primer set used for genotyping and detection of virulence genes of *S. aureus* (Table-2).

**Table-2:** Primers list, program and protocol of each primer.

Primer	Program						Protocol

	All primers were synthesized by Sigma-Aldrich, USA							

	Initial denaturation	No. of cycles	Denaturation	Annealing	Extension	Final extension	
(1) 16 S *rRNA*	-	30	95°C for 1 min	70°C for 45 s	72°C for 1 min	72°C for 10 min	(1)
(2) *PVL*	-	30	94°C for 30s	55°C for 30s	72°C 1 min	72°C for 10 min.	(1)
(3) *CLFA*	-	35	94°C for 60 sec	57°C for 60 sec	72°C for 1 min	72°C for 10 min.	(1)
(4) *Coa*	95°C for 2 min	30	95°C 30s	58°C for 2 min	72°C for 4 min	72°C for 7 min	(1)
(5) *Hla* and (6) *Hlb*	95°C for 5 min	30	94°C for 60 sec	55°C for 30 s	72°C for 1 min	72°C for 10 min.	(1)
(7) Enterotoxins	-	25	94°C for 30 s	50°C for 30 s	72°C for 30 s	72°C for 2 min	(2)
Protocol (1)	PCR was performed in a 50μl reaction mixture containing 2 μl of template DNA (approximately 500 ng/μl), 5 μl of ×10 PCR buffer (750 mM Tris–HCl (pH 8.8), 200 mM (NH_4_)_2_SO_4_, and 0.1% Tween 20), 200 μM of each of the four deoxynucleotide triphosphates, 1 U of Taq DNA polymerase (Roch Applied Science), and 50 pmol of each primert						
Protocol (2)	PCR was performed in a 50μl reaction mixture containing 2 μl of template DNA (approximately 500 ng/μl), 5 μl of ×10 PCR buffer (750 mM Tris–HCl (pH 8.8), 200 mM (NH_4_)_2_SO_4_, and 0.1% Tween 20), 200 μM of each of the four deoxynucleotide triphosphates, 1 U of Taq DNA polymerase (Roch Applied Science), and 50 pmol of each primer						

Hlb=Betahemolysin, Hla=Alphahemolysin, Coa=Coagulase, CLFA=Clumping factor A, PVL: Panton valentine leukocidin, PCR: Polymerase chain reaction

## Results

Table-3 shows results of tested animals, CM, obtained isolates, identified *S. aureus*, hemolytic activity, and thermo nuclease production.

**Table-3:** Results of tested animals, CM, obtained isolates, identified *S. aureus*, hemolytic activity, and thermo nuclease production.

Type of test	Clinically mastitic cases			Subclinically mastitic cases		
	
	Total no.	Cattle	Buffaloes	Total no.	Cattle	Buffaloes
	
	143	94	49	59	35	24
CMT^a^	123	86/94 (91.49%)	37/49 (75.51%)	24	13/35 (37.14%)	11/24 (45.83%)
Obtained isolates on specific media	38	28/86 (32.56%)	10/37 (27%)	21	10/13 (76.92%)	11/11 (100%)
Biochemically identified as *S. aureus*	16	11/28 (39.29%)	5/10 (50%)	16	8/10 (80%)	8/11 (72.73%)
Hemolytic activity						
Alpha α	16 (100%)	4/11 (36.4%)	1/5 (20%)	9 (56.25%)	2/8 (25%)	4/8 (50%)
Beta β		7/11 (63.6%)	4/5 (80%)		3/8 (37.5%)	0 (0.0%)
Thermo nuclease production	4/16 (25%)	4/11 (36.36%)	0 (0.0%)	9 (56.25%)	5/8 (62.5%)	4/8 (50%)

CM=California mastitis, *S. aureus=Staphylococcus aureu*s, CMT=California mastitis test

Table-4 shows confirmation of S. *aureus* isolates from cattle and buffaloes and molecular detection of some virulence factors by PCR using 16S *rRNA*, PVL, CLA coagulase gene, Hla, Hlb, and primers targeting genes of enterotoxins.

**Table-4:** Confirmation of *S. aureus* isolates from cattle and buffaloes and molecular detection of some virulence factors by PCR using 16S *rRNA*, *PVL*, *CLA* coagulase gene, *Hla*, *Hlb*, and primers targeting genes of enterotoxins.

Type of animal	No. of animals and isolates	Type of mastitis	PCR using primers to detect						

			16S *rRNA* (%)	*PVL* (%)	*CLA* (%)	*Coa* Gene (%)	*Hla* (%)	*Hlb* (%)	Enterotoxins targeting genes
Cattle	11	Clinical	11/11 (100)	6/11 (54.54)	6/11 (36.4)	11/11 (100)	4/11 (36.4)	7/11 (63.6)	-ve
	8	Subclinical	8/8 (100)	7/8 (87.5)	3/8 (37.5)	8/8 (100)	2/8 (25)	3/8 (37.5)	4/8 (50%) Type D toxin
Buffaloes	5	Clinical	5/5 (100)	5/5 (100)	4/5 (80)	5/5 (100)	1/5 (20)	4/5 (80)	-ve
	8	Subclinical	8/8 (100)	3/8 (37.5)	3/8 (37.5)	8/8 (100)	4/8 (50)	0 (0.0)	-ve
Total	32		32/32 (100)	21/32 (65.6)	16/32 (50)	32/32 (100)	11/32 (34.4)	14/32 (43.75)	4/32 (12.5%)

Hlb=Betahemolysin, Hla=Alphahemolysin, Coa=Coagulase, CLFA=Clumping factor A, PVL: Panton valentine leukocidin, PCR: Polymerase chain reaction, *S. aureus=Staphylococcus aureus*

### Statistical analysis

The prevalence to every test was calculated as the number of positive cattle divided by the number of examined cases within the specified period.

The Pearson and McNamara’s Chi-square tests were respectively used to estimate the association between the CMT, the culture results (isolation and identification), phenotypic virulence factors and to compare the phenotypically identified isolates with the genotypic molecular methods using SPSS statistic software version 17.0.

## Results and Discussion

Concerning results obtained from Table-3 CMTpositive prevalences of clinically mastitic cases were91.48% and 75.51% for cattle and buffaloes, respectively, as well as of subclinical mastitic cases 37.14% and 45.83% for cattle and buffaloes, respectively. The obtained high results of CMT in clinically mastitic cattle was 91.48% gave great agreement with Joshi and Shrestha [17]. While, CMT of apparently healthy cattle indicating subclinical mastitis the prevalence was 37.14% which is nearly similar to Mahmoud [18]. The gained CMT and clinical mastitis results for buffaloes was 75.51% nearly similar to Linda *et al*. [19] who found that prevalence of clinically mastitic buffaloes in hygienically untrained households was 60.4%. Whilst, CMT of apparently healthy buffaloes gave 45.83% indicating subclinical mastitis this result was nearly similar to Akhtar *et al*. [20]. Commenting on the isolation and identification performed in Egypt, results obtained from Table-3 was conspicuous that S. *aureus* was higher in clinically mastitic buffaloes than cattle with prevalences of 50% and 39.29%, respectively. Whereas, identified S. aureus was higher in subclinically mastitic cattle than buffaloes with prevalences of 80% and 72.73%, respectively. It was confirmed in Egypt that S. *aureus* considered the predominant among mastitis causing pathogens followed by Streptococcus agalactiae [21] and *Escherichia coli* [22]. The identified S. *aureus* prevalence from clinically mastitic buffaloes was nearly similar to Hameed [23] who noted that the prevalence of clinical bovine mastitis caused by S. *aureus* was 53.85% in buffaloes from Tehsil Burewala Pakistan. However our result concerning the identification of S. *aureus* from clinically mastitic cattle 39.29% and from subclinical mastitic cattle and buffaloes 80% and 72.73% coincides with Hase *et al*. [24].

**Figure-1:**
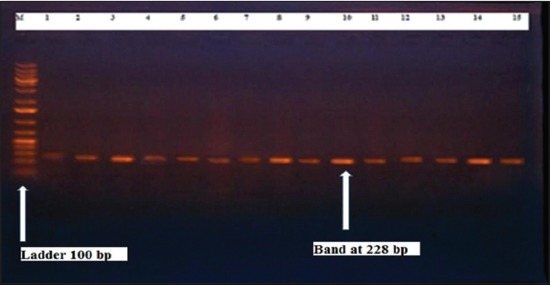
Results of molecular detection of *Staphylococcus aureus* 16S rRNA where (M is marker of 100 bp range, while lanes from (1 to 32) indicate positive isolates and result appear at 228 bp, moreover, lanes (33 and 34) represent control positive and control negative respectively.

**Figure-2:**
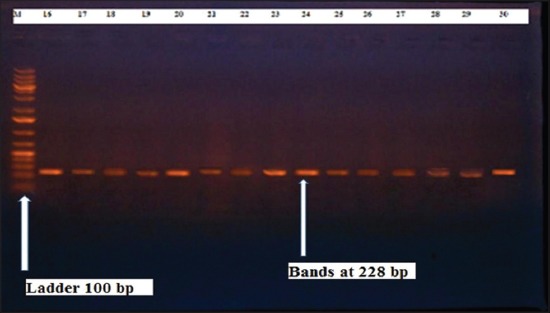
Results of molecular detection of Panton valentine leukocidin using where (M; is marker of 100 bp range, while lanes from (1 to 21) indicate positive isolates and result appear at 433 bp, moreover, lanes (22 and 23) represent control positive and control negative respectively.

**Figure-3:**
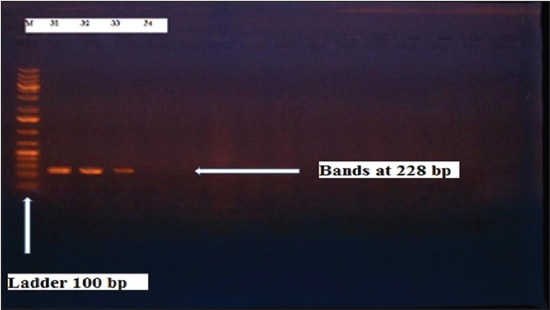
Results of molecular detection of Clumping factor A using where (M; is marker of 100 bp range, while lanes from (1 to 16) indicate positive isolates and result appear at 980 bp, moreover, lanes (17 and 18) represent control positive and control negative respectively.

**Figure-4:**
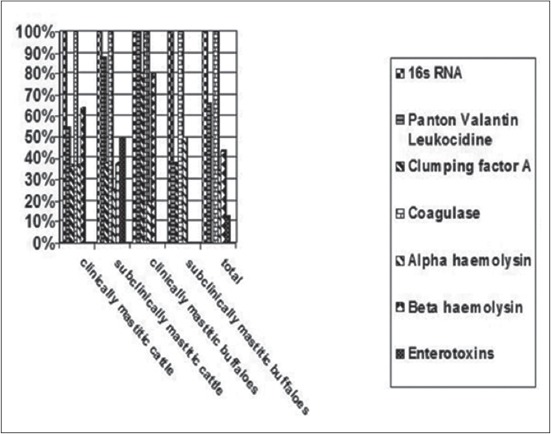
Collective results of genotypic detection of *Staphylococcus aureus* virulence factors.

**Figure-5:**
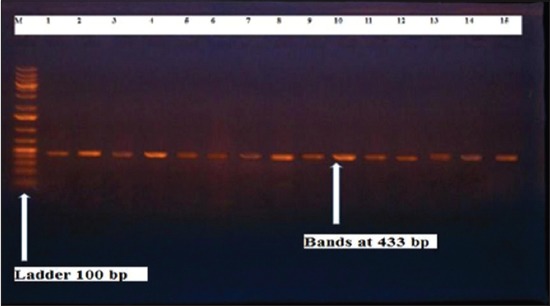
Results of molecular detection of Coa gene where (M; is marker of 100 bp range, while lanes from (1 to 32) indicate positive isolates and result appear at 410 bp, moreover, lanes (33 and 34) represent control positive and control negative respectively.

**Figure-6:**
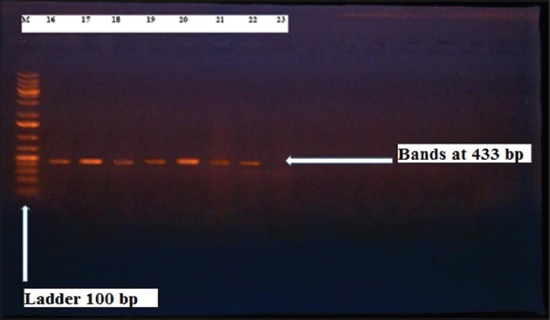
Results of molecular detection of Hla gene where (M; is marker of 100 bp range, while lanes from (1 to 11) indicate positive isolates and result appear at 209 bp, moreover, lanes (12 and 13) represent control positive and control negative respectively.

**Figure-7:**
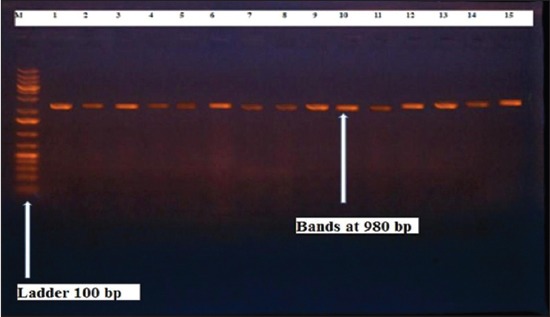
Results of molecular detection of Hlb gene where (M; is marker of 100 bp range, while lanes from (1 to 14) indicate positive isolates and result appear at 309 bp, moreover, lanes (15 and 16) represent control positive and control negative respectively.

**Figure-8:**
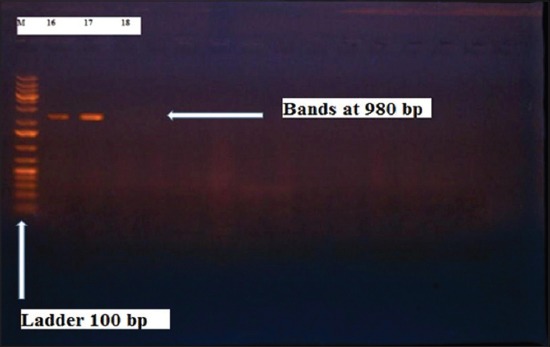
Results of molecular detection of Enterotoxine D gene where (M; is marker of 100 bp range, while lanes from (1 to 4) indicate positive isolates and result appear at 309 bp, moreover, lanes (5 and 6) represent control positive and control negative respectively.

**Figure-9:**
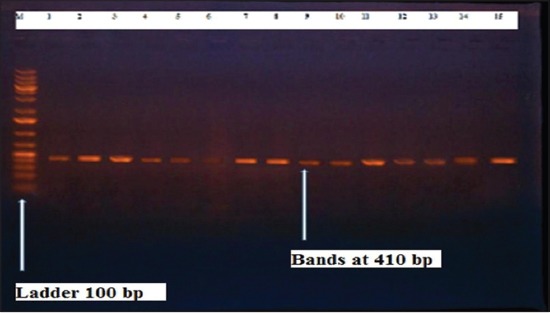
Collective results of genotypic detection of *Staphylococcus aureus* virulence factors.

**Figure-10:**
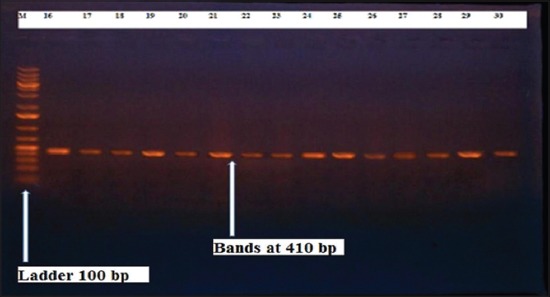
Results of molecular detection of Coa gene where (M; is marker of 100 bp range, while lanes from (1 to 32) indicate positive isolates and result appear at 410 bp, moreover, lanes (33 and 34) represent control positive and negative control respectively.

**Figure-11:**
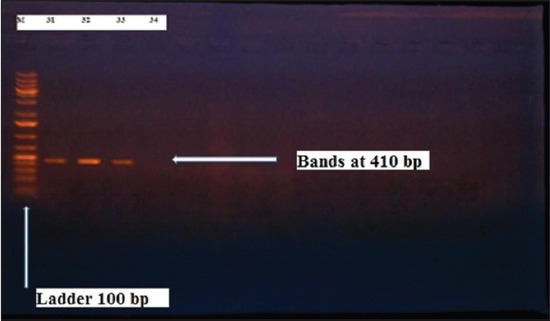
Results of molecular detection of Coa gene where (M; is marker of 100 bp range, while lanes from (1 to 32) indicate positive isolates and result appear at 410 bp, moreover, lanes (33 and 34) represent control positive and negative control respectively.

**Figure-12:**
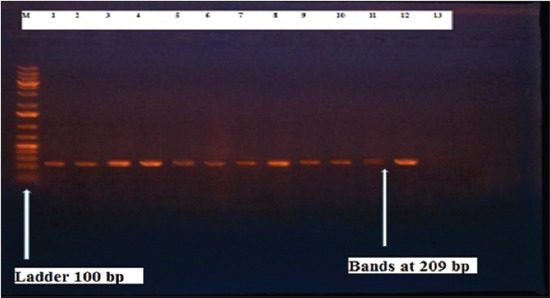
Results of molecular detection of HIa gene where (M; is marker of 100 bp range, while lanes from (1 to 14) indicate positive isolates and result appear at 309 bp, moreover, lanes (15 and 16) represent control positive and negative control respectively.

**Figure-13:**
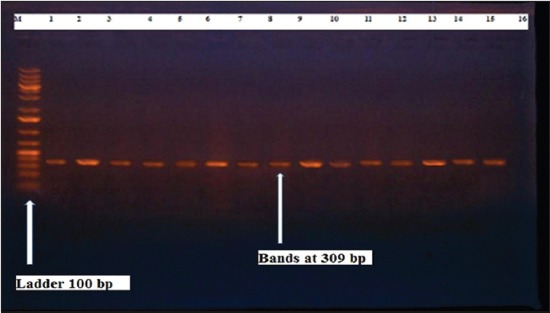
Results of molecular detection of HIb gene where (M is marker of 100 bp range, while lanes from (1 to 14) indicate positive isolates and result appear at 309 bp, moreover, lanes (15 and 16) represent control positive and control negative respectively.

**Figure-14:**
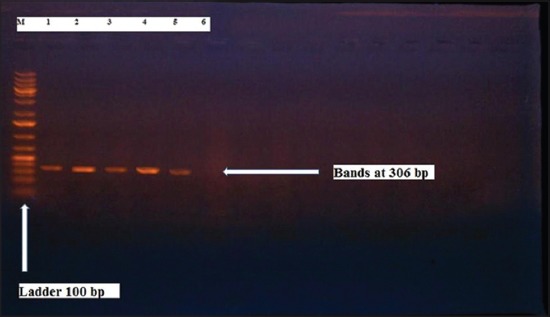
Results of molecular detection of Enterotoxine D gene where (M; is marker of 100 bp range, while lanes from (1 to 4) indicate positive isolates and result appear at 306 bp, moreover, lanes (5 and 6) represent control positive and control negative respectively.

Herein, from Table-3, it is overt that the total hemolytic activity of *S. aureus* isolated from clinically mastitic cases was assessed and all the isolates 100% were completely hemolytic, α hemolysis results were 36.4% and 20% for cattle and buffaloes respectively. In addition to that, β hemolysis results were 63.6% and 80% for cattle and buffaloes respectively. Furthermore, the total hemolytic activity of subclinical mastitic *S. aureus* was 56.25%, α hemolysis results were 25% and 50% for cattle and buffaloes, respectively. Moreover, β hemolysis results were 37.5% and 0.0% for cattle and buffaloes respectively. Herby, the higher total hemolytic activity of *S. aureus* from clinically mastitic cases 100% [25]. Results obtained in Table-3 express the result of (TNase) produced from isolated *S. aureus* as follows total result of positive (TNase) production from clinically mastitic cases was 25%, but the result of subclinically mastitic cases was 56.25% our obtained results were lower than that obtained by Hamama [26]. Referring to results of Table-4 and Figures 1-4 which indicate implementation of simplex PCR for confirmation of isolates using 16S *rRNA* which proved that 100% of the isolates were confirmed to be *S. aureus* and that concurred with Monday and Bohach [11]. Focusing on detection of PVL and results of Table-4 and Figures-2-6 only 65.6% of the isolates proved the existence of PVL and that nearly similar to Unal *et al*. [27]. Also, putting in consideration the results of *CLFA* as expressed in Table-4 and Figures-4, -7 and -8, 14 about 46.9% of isolated *S. aureus* harbored *CLFA* that was nearly similar to results obtained by Momtaz *et al*. [28]. Concerning results in Table-4 and Figures-4, and -9-11 of simplex PCR for detection of coagulase gene *Coa* all isolates 100% harbored this gene this result mainly agree with the result recorded by Karakulska *et al*. [29]. It is apparent from Table-4 and Figures-4, -12, and -13) that there located genetic confirmation of the phenotypic hemolytic results, performed by testing the presence of *Hla* and *Hlb* genes and the existence rates were 34.4% and 43.75% within the isolates, respectively that’s basically concurred [30]. Some *Staphylococci* produce staphylococcal enterotoxins (*SEs*) involved in staphylococcal food poisoning syndrome in humans, especially in the TSST-1, the *ETA* and *ETB* that cause staphylococcal scalded skin syndrome in children and newborns. Recently, 19 serologically distinct SEs have been identified. SEs A, B, C, D, and E are the classical five major types. However, other new enterotoxins have been described by Thomas *et al*. [31]. Because of the importance of these toxins in the public health and food sectors, an efficient screening to detect the prevalence of enterotoxigenic strains in foods is required, so we used multiplex PCR technique for detection of enterotoxigenic *S. aureus*. From Table-4 and Figures-4 and -14 only 12.5% of the tested isolates were positive for enterotoxine D, and that mainly coincides with Hamama [26]. Hence, the genotypic results of this study might help to better understand the prevalence and distribution of *S. aureus* clones among bovines and will help to assess control strategies for *S. aureus* infections [32].

## Conclusion

It is concluded that simplex and multiplex PCR assays can be used as rapid and sensitive diagnostic tools to detect the presence of *S. aureus* and characterize its virulence factors that help in detection of severity of infection, distribution and stating preventive and control strategies.

## Authors’ Contributions

MSE carried out the laboratory work, helped in isolation and identification of isolates, made all the molecular steps, compiled and analyzed information and data, wrote and drafted the manuscript, AEME revised the manuscript and MAD helped in sampling, isolation and identification of isolates.
